# Exploring Smart Health Wearable Adoption Among Singaporean Older Adults Based on Self-Determination Theory: Web-Based Survey Study

**DOI:** 10.2196/69008

**Published:** 2025-03-19

**Authors:** Hyunjin Kang, Tingting Yang, Nazira Banu, Sheryl Wei Ting Ng, Jeong Kyu Lee

**Affiliations:** 1 Wee Kim Wee School of Communication and Information, Nanyang Technological University, 31 Nanyang Link, Singapore, 637718, Singapore, 65 6908-3431; 2 Department of Health and Exercise Science, University of Oklahoma, Norman, OK, United States

**Keywords:** smart health wearables, self-determination theory, AI anxiety, perceived privacy risk, health consciousness

## Abstract

**Background:**

Smart health wearables offer significant benefits for older adults, enabling seamless health monitoring and personalized suggestions based on real-time data. Promoting adoption and sustained use among older adults is essential to empower autonomous health management, leading to better health outcomes, improved quality of life, and reduced strain on health care systems.

**Objective:**

This study investigates how autonomy-related contextual factors, including artificial intelligence (AI) anxiety, perceived privacy risks, and health consciousness, are related to older adults’ psychological needs of competence, autonomy, and relatedness (RQ1). We then examined whether the fulfillment of these needs positively predicts older adults’ intentions to adopt these devices (H1), and how they mediate the relationship between these factors and older adults’ intentions to use smart health wearables (RQ2). Additionally, it compares experienced and nonexperienced older adult users regarding the influence of these psychological needs on use intentions (RQ3).

**Methods:**

A web-based survey was conducted with individuals aged 60 years and above in Singapore, using a Qualtrics survey panel. A total of 306 participants (177 male; mean age of 65.47 years, age range 60‐85 years) completed the survey. A structural equation model was used to analyze associations among AI anxiety, perceived privacy risks, and health consciousness, and the mediating factors of competence, autonomy, and relatedness, as well as their relationship to smart health wearable use intention.

**Results:**

Health consciousness positively influenced all intrinsic motivation factors—competence, autonomy, and relatedness—while perceived privacy risks negatively affected all three. AI anxiety was negatively associated with competence only. Both privacy risk perceptions and health consciousness were indirectly linked to older adults’ intentions to use smart health wearables through competence and relatedness. No significant differences were found in motivational structures between older adults with prior experience and those without.

**Conclusions:**

This study contributes to the application of self-determination theory in promoting the use of smart technology for health management among older adults. The results highlight the critical role of intrinsic motivation—particularly competence—in older adults’ adoption of smart health wearables. While privacy concerns diminish motivation, health consciousness fosters it. The study results offer valuable implications for designing technologies that align with older adults’ motivations, potentially benefiting aging populations in other technologically advanced societies. Developers should focus on intuitive design, transparent privacy practices, and social features to encourage adoption, empowering older adults to use smart wearables for proactive health management.

## Introduction

### Background

Smart health wearables are devices powered by artificial intelligence (AI) designed to be worn on the body, enhancing health management. This technology holds significant promise for the older population, offering a nonintrusive way to continuously monitor health information and provide valuable assistance [1,2]. The use of smart health wearables has been verified in clinical intervention research and proven to assist in self-health management and prevention of chronic diseases, such as diabetes, hypertension, and dementia [3]. With technological advancements today, some of these technologies have already been integrated into personal devices, such as smartphones and other mobile devices, making it easier for older adults to track their health conveniently [4]. To fully leverage the potential of health wearables, it is essential to promote the adoption and sustained use among older adults, empowering them in autonomous health management. This can lead to better health outcomes, an improved quality of life, and reduced strain on the health care system [5].

The promotion of adoption and sustained use of smart health wearables can be understood through the lens of self-determination theory (SDT) [6,7], which posits that fostering intrinsic motivation, rather than extrinsic, is essential for encouraging prolonged active engagement in a behavior. SDT suggests that fostering an environment which supports an individual’s self-determination in engaging in a specific behavior is crucial for satisfying fundamental human needs that drive intrinsic motivation [6]. We examine AI anxiety, privacy risk perceptions, and health consciousness as the critical contextual factors shaping self-determination of using smart health wearables. These 3 contextual factors are closely tied to key smart health wearable aspects. (1) AI anxiety [8] stems from the automated, AI-driven recommendations in smart health wearables, which can make users feel they are losing control over health decisions. (2) Privacy risk perceptions [9] relate to the continuous data collection by smart health wearables; if inadequately protected, users’ sense of control over their personal information can be reduced. (3) Health consciousness aligns with smart health wearables’ purpose of empowering users to take charge of their health, reinforcing autonomy for proactive self-management [10].

Another key proposition of SDT is that fulfilling the 3 psychological needs of competence, relatedness, and autonomy is essential for intrinsic motivation [6]. When applied to smart health wearables for older adults, the extent to which these devices help older adults feel capable, enable independent choices, and foster a sense of connection with others can shape their intrinsic motivation to engage with the devices and promote sustained usage.

Taken together, this study explores how 3 contextual factors related to self-determination in using smart health wearables—AI anxiety, privacy risk perceptions, and health consciousness—are associated with fulfilling the needs for competence, relatedness, and autonomy, thereby promoting use intentions. Additionally, the study examines whether differences exist between experienced and nonexperienced wearable users in terms of the psychological needs influencing their intentions. Understanding these distinctions provides insight into the motivators for each group at different adoption stages, offering evidence for targeted strategies to sustain user engagement among older adults and thereby promote continuous, effective self-health management [11].

Through this study, we aim to contribute to the literature on self-determination in health promotion through smart technology by identifying key factors that intrinsically motivate older adults to use smart health wearables, which ultimately enhances their long-term well-being. A web-based survey has been conducted in Singapore, one of the world’s most connected nations with high information and communication technology penetration and a robust AI ecosystem [12]. Furthermore, Singapore has recently become a “super-aged society,” with nearly one-fifth of its population over 65 years old [13]. As such, the insights gained from this research can offer valuable, generalizable implications for designing technologies that align with older adults’ motivations, potentially benefiting aging populations in other technologically advanced societies.

### Literature Review: Smart Health Wearables for Older Adults’ Self-Health Management

Wearables refer to “intelligent computing devices integrated into various accessories, including clothing, fashion accessories, and other everyday items worn by consumers” [14]. Smart health wearables are wearable devices designed to enhance users’ health management, offering users a seamless and integrated user experience. Smart health wearables have many benefits, with their main benefit being empowering older adults to take charge of their health [15]. By integrating sensors, (eg, bio, motion, and environmental sensors), internet connection, as well as AI, smart health wearables enable individual users to track and exchange data, and even make smart and personalized decisions for their health management [1]. Some examples of smart health wearables include biosensors, GPS, and radio frequency identification (RFID) technologies that can monitor health conditions by tracking physical information such as blood pressure, oxygen level, and sleep patterns to provide personalized health feedback and medical suggestions. This feedback is valuable for those managing chronic conditions, such as diabetes or cardiovascular diseases [16]. New and improved wearable designs (eg, smartwatches) incorporate advanced algorithms and machine learning to analyze users’ data and provide personalized workout programs and nutrition recommendations [17].

The potential of health wearables to improve self-health management is increasingly recognized. However, adoption rates among older adults remain low, largely due to skepticism about the tangible benefits of using wearables [18,19]. Despite a growing body of research exploring enablers and barriers to adopting smart health wearables, the literature remains fragmented [20]. Further studies are needed to understand how these devices are perceived and what motivates older adults to adopt them long-term. For example, recent studies have reported that older adults are more likely to adopt technology if they perceive a clear and immediate benefit from its usage. If the advantages of wearables are not clearly communicated or experienced, older adults may not see them as worth the investment [21]. However, such insights only scratch the surface. More in-depth exploration is necessary to uncover intrinsically motivated factors that can help older adults perceive a clear benefit from adopting wearables. Understanding these factors is crucial to fostering long-term engagement with these wearable devices. This study draws upon SDT and the motivational technology model to identify intrinsic motivators that can encourage older adults to adopt smart health wearable devices for their self-health management.

### Three Contextual Factors Related to Autonomous Motivation

#### Overview

One of the key propositions of SDT is that creating an environment that supports one’s self-determination in engaging in a given behavior is essential for fulfilling the key human needs required for fostering intrinsic motivation. In environments that respect and encourage individuals’ autonomy, they are more likely to develop a sense of their true selves and achieve self-determination for their actions [6]. In social conditions that pressure people’s autonomous behavior, autonomy is deprived. In such situations, the fulfillment of the 3 needs for intrinsic motivation will be hampered. However, the key human needs for intrinsic motivation will be nurtured in autonomy-supportive contexts, where the external environment supports one’s self-initiation and choice.

The role of autonomous motivation in encouraging behavior is particularly significant in health-specific contexts. Being in an autonomy-supportive health care climate, for instance, and having autonomous motivation are linked to successful physical activity and dietary management, which, in turn, leads to better health outcomes for patients with type 2 diabetes [22]. Likewise, feelings of autonomous motivation positively impacted the behavioral intentions and usage behaviors of sports apps among students, contrary to feelings of controlled motivation [23]. However, solely using smart health wearables is insufficient to encourage self-health management. A study that sought to promote physical activity among adolescents using health wearables found that, contrary to expectations, feelings of autonomous motivation decreased over an 8-week period because of feelings of peer comparison that created a social environment where participants’ autonomy was undermined [24]. This highlights the importance of contextual factors in shaping autonomous motivation to use smart health wearables for health management. Moreover, the technology acceptance model (TAM) [25] and the unified theory of acceptance and use of technology (UTAUT) [26] explain how users accept a given technology. These theories suggest that external factors, such as social influence, should be considered to understand people’s attitudes and intentions to use technology.

This study examines the roles of 3 social-contextual factors—AI anxiety, perceived privacy risks, and health consciousness—that are crucial for creating a context that fosters the autonomy of older adults in engaging in self-health management using smart health wearables.

#### AI Anxiety

There is growing tension between human agency and machine agency in the context of the rise of AI, especially when user experience relies on the algorithms of smart technology [27]. Deci and Ryan [28] defined human agency as motivated behaviors that emanate from one’s integrated self. In the context of communication with smart technology, user agency is defined as “the degree to which the self feels like that he or she is a relevant actor” and a feeling that the technology provides “manipulability” for the user to exercise their influence throughout the interaction [29, p. 61]. The feeling of being in control is essential as it bolsters people’s intrinsic motivation.

However, as AI techniques advance at an unprecedented speed, people may begin to experience negative psychological tension driven by their natural desire to limit AI agency [27]. AI anxiety describes the psychological status of fear and trepidation when people are concerned that they will lose autonomy and be controlled by AI [8]. It is caused by the constantly evolving technological advancement of AI, along with the challenges and uncertainty that AI brings [30]. For example, Airbnb hosts experienced this algorithm-related AI anxiety as they navigated a complex ecosystem filled with uncertainty and frustration, since the algorithms comprise both “known and unknown factors” [31, p. 9].

While existing research indicates the prevalence of AI anxiety across various populations [32], AI anxiety may be particularly prominent among older adult users. Research shows that age is positively associated with greater anxiety over computers [33]. Although there are ontological differences between traditional computing technologies and AI, AI technologies may generate a wider range of anxieties because of their complexity and perceived autonomy [34]. Furthermore, low levels of technological literacy among older adults may worsen AI anxiety since a key dimension of AI literacy involves the ability to learn AI, which can be particularly challenging for older adults [34,35].

AI anxiety becomes even more pronounced when AI is used to deliver health care services, like in the case of smart health wearables. The decision-making processes of AI models that directly affect the user’s well-being may be beyond the understanding of users [36]. Studies have highlighted the fear and frustration expressed by older adults when using smart health wearables to monitor their blood oxygen saturation levels [37], which illustrates how smart health wearables can deepen pre-existing anxieties. Since studies indicate that technological anxiety can negatively affect attitudes and intentions to use technology [3,38,39], we investigate AI anxiety as a significant factor shaping intrinsic motivation for using smart health wearables.

#### Perceived Privacy Risks

Privacy risks, throughout the various stages of designing, distributing, and using smart health wearables, can cause privacy violations. Technically, (1) most wearable devices are equipped with various sensors (eg, motion, location, and physiological sensors) that continuously collect data, (2) these data are highly personal and thus sensitive, and (3) opportunistic data usage can lead to privacy breaches [40]. Hence, using smart health wearables for health management inevitably triggers people’s concerns about smart health wearables’ ability to protect privacy. Complicated terms regarding data collection and sharing further exacerbate this concern, with many users unaware of how their data are managed [18,41]. Additionally, incidents involving unauthorized access and cyberattacks compound these concerns [40]. Particularly for older adult users, concerns about privacy and data security are heightened [9].

Moreover, privacy is essential for developing an autonomous self. It is commonly accepted that the human body and health data are “the central mediator of autonomy and individual privacy” [42, p. xvi]. Therefore, individuals’ controllability over their information [43] or “selective control of access to the self or to one’s group” [44, p. 18] is the core element of privacy. Through such processes of boundary regulation, individuals decide how open they want to be to others. However, smart health wearables need health-related personal information to function (eg, customize exercise plans and make smart recommendations). Hence, there exists this tension between (1) the need to grant controlling power of private information to smart health wearables and (2) people’s need for autonomous decision-making about information disclosure. For example, when using smart home applications integrating sensors and other network technologies, older adult users expressed their concern about their controllability over their privacy; however, this sense of tension can be ameliorated if they have “full control of the sensor,” meaning they would feel more in control if they have a say about when to activate or deactivate the device [45, p. 4750]. Thus, perceived privacy risks are closely tied to feelings of autonomy, and the control of personal information is key to intrinsic motivation for using health care wearables [46].

#### Health Consciousness

Health consciousness refers to how individuals care about their health and the level at which they are motivated to engage in preventive health practices such as health information seeking, home-based exercise, and healthy food choices [47]. Specifically, health consciousness reflects people’s orientation toward three health-related dimensions: (1) personal health awareness, (2) self-responsibility, and (3) health motivation [48]. Health consciousness has repeatedly been shown to have a positive effect on health-related behaviors like using the internet to search for health information [10], choosing foods based on their health benefits [47], or using dietary and fitness apps [49].

There are several reasons to explain why health consciousness motivates older adults to engage in self-health management. Health-conscious people tend to have a stronger belief in their ability to control their health, which influences their decisions to engage in health-promoting activities [50]. Furthermore, health consciousness can motivate people to be proactive in looking for ways to evaluate their conditions, like taking up information and communication technology to mitigate loneliness [51]. Proactiveness is a distinct characteristic of health consciousness and differs from simply reacting to negative health outcomes. This aligns with research using the health belief model, which suggests that health-related internet use was more strongly proactively motivated by health consciousness, rather than being influenced by perceptions of one’s health risk in a reactive manner [10]. Smart health wearables that are designed to enable self-directed health management activities [52] precisely meet the needs of health-conscious individuals. Thus, it would be fruitful to assess health consciousness as a potential factor encouraging the adoption of smart health wearables among older adults.

### Three Basic Needs for Self-Determination

We used the SDT [6,7] to examine the factors related to the motivation for adopting smart health wearables among older adults. The core idea of SDT is that intrinsic motivation is necessary for sustaining engagement in a particular behavior. The 3 basic needs—competence, relatedness, and autonomy—are fundamental sources of intrinsic motivation and the natural tendency for self-development.

Competence refers to the ability to achieve personal goals. Autonomy is the freedom to make self-initiated choices, and relatedness reflects feeling connected and cared for by social groups or communities. When individuals perceive that a particular behavior fulfills these 3 basic needs, they tend to find it enjoyable (ie, intrinsically motivating) and will engage in it without relying on external rewards. Hence, from the lens of SDT, this study examines how the 3 psychological needs shape intrinsic motivation and ensures the long-term use of smart health wearables for older adults’ self-health management.

As with other interactive technologies, design elements in smart health wearables can influence the sense of competence, autonomy, and relatedness through their intelligent, user-focused features [53]. For example, features such as goal setting and reward badges can enhance users’ competence by providing feedback on their achievements and progress. Yet, older adults often struggle with setting up or understanding the data generated by these devices, which can undermine their sense of competence [54]. Additionally, while personalized nudges and recommendations from these devices may sometimes compromise users’ sense of autonomy by making decisions based on their data [27], customizable interfaces can empower users to feel more in control. Furthermore, social features—such as communities, friend invitations, and participation in challenges—can enhance a sense of relatedness among users [24,53]. Wu and Lim [21] found that older adults are more likely to use wearables when family members or friends in their close social circle also adopt the devices.

Overall, this study explores 3 social-contextual factors influencing the autonomous use of smart health wearables for health management—AI anxiety, perceived privacy risks, and health consciousness—and their potential associations with the psychological needs related to intrinsic motivation among older adults. Accordingly, we pose the following research question:

**RQ1:** How are autonomy-related contextual factors regarding smart health wearable use (ie, AI anxiety, perceived privacy risks, and health consciousness) associated with the sense of competence, autonomy, and relatedness among older adults?

In addition, we aim to unpack the relationship between the psychological needs of older adults and their intention to use smart health wearables. Self-determined motivation shaped by the fulfillment of these 3 needs plays a crucial role in people’s intention to use technology [55]. For instance, a study found that intentions to use health technology, such as wellness clouds for health tracking, were positively associated with intrinsic motivation, driven by expectations of performance, playfulness, and ease of use [56]. Thus, we hypothesize:

**H1:** Perceived competence, autonomy, and relatedness in using smart health wearables will be positively associated with older adults’ intentions to use these devices.

We expect that the needs of competence, autonomy, and relatedness fulfilled by health wearables use would mediate the relationships between the autonomy-related contextual factors and use intention. This study will explore these indirect associations by proposing the following research question:

**RQ2:** How do perceived competence, autonomy, and relatedness in using smart health wearables mediate the relationships between the autonomous-related contextual factors (ie, AI anxiety, perceived privacy risks, and health consciousness) and older adults’ intentions to use these devices?

### Experienced Versus Nonexperienced Users

We also examine potential differences in motivation between older adults with and without prior experience using smart health wearables. According to the UTAUT and TAM models, as discussed earlier, users’ prior experience with using the technology is also one of the key variables, as prior experience can work as a conditional factor in changing the degree of effect of other antecedent factors on technology acceptance (eg, intention to use and attitudes toward a technology) [26]. Specifically, whether a user has experience using a technology may affect their evaluation of motivations, effort expectancy, social influence, and perceived ease of use, among other factors [26,57]. For example, one study about smart health wearables found that the relation between behavior intention and actual use is stronger for more experienced users, compared with the new users; in addition, the experienced users rated the facilitating conditions (eg, resource and knowledge they received) as less important [58]. Interestingly, among older adults, whether users have prior experience also showed a difference in what types of smart health wearables they choose: compared with experienced users whose choice focuses mostly on smartwatches and wristbands, nonexperienced users’ choices were more dispersed, showing a wide variety of preferences such as smart rings and clips among others. This indicates that prior experience might affect users’ overall expectations among many technology acceptance indicators [59], supporting the proposition that prior experience may be a conditional factor that differentiates users’ motivational structure predicting their technology adoption intentions [60], as experienced users tend to exhibit greater confidence and skill in navigating wearable features [61,62]. Experienced older adults may more readily recognize benefits such as improvements in health metrics or physical activity [16]. Conversely, older adults with limited or no prior experience may have different expectations regarding wearables, often accompanied by uncertainty. While such uncertainty can foster positive expectations about the benefits of devices, it may also provoke feelings of intimidation. Older adults without prior experience may struggle to fully grasp the benefits of smart health wearables early on. Moreover, without clear evidence of the device’s impact on their well-being, these older adults might question the use of the technology, resulting in lower intrinsic motivation to adopt such devices [63].

However, the moderating effect of prior experience from existing literature shows inconsistent effects. For example, one study examined whether users’ prior experience of using robots predicts their approval of autonomous delivery robots, and the correlation was not significant [60]. In light of these discrepancies, we will examine whether and how these 2 groups—those with and without prior experience—differ in their motivational structures for using smart health wearables in health management. Understanding the motivational differences between those who are experienced versus not experienced users of smart health wearables offers valuable insights into the distinct needs of users in different adoption stages. These insights can guide the development of more user-friendly designs and tailored educational strategies, ultimately encouraging wider adoption among hesitant or inexperienced individuals. Accordingly, we propose the following research question:

**RQ3:** How are experienced older adult users different from the nonexperienced older adult group regarding the relationship between the 3 psychological needs and use intention?

**Figure 1.**
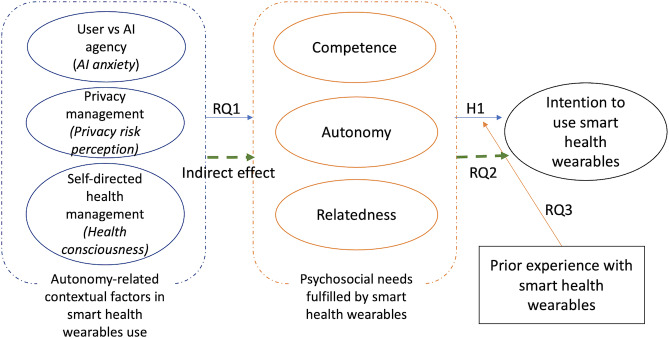
Hypothesized model summary. AI: artificial intelligence; H: hypothesis; RQ: research question.

Figure 1 summarizes the model tested in this study.

## Methods

### Data Collection

A web-based survey was conducted with individuals over the age of 60 years in Singapore from March to April 2022. For data collection, we used a survey panel from Qualtrics, one of the major global survey companies, which provides panels across various age groups in different countries. In total, 306 participants (177 male; mean age of 65.47 years, age range 60‐85 years; ethnicity: 84.6% [259/306] Chinese, 3.3% [10/306] Malay, 6.5% [20/306] Indian, and 5.6% [17/306] others) completed the survey. At the beginning of the study, they were given descriptions and examples of smart health wearables. Then, they were asked if they had used such devices before. Of the 306 participants, 163 (53.3%) responded “yes” indicating their prior experiences with smart health wearables, and fitness trackers were the most common type of wearable used (135/306, 44.1%). Meanwhile, 143 (46.7%) responded “no” to the question asking about their prior experience.

### Ethical Considerations

The survey procedure and materials were reviewed and approved by the institutional review board at Nanyang Technological University (IRB-2022-163). Informed consent was obtained before participants began the survey, and they had the option to opt out. All collected data were deidentified. Compensation was provided to participants by the survey company in the form of credit points in accordance with its policy.

### Measurement

#### AI Anxiety

To measure the anxiety caused by uncertainty about how AI works, 3 items were adapted from Meuter et al [64], originally developed for measuring user anxiety about service technology. A 7-point Likert scale was used (1=strongly disagree to 7=strongly agree; eg, “I feel apprehensive about using AI technology”).

#### Perceived Privacy Risk

Perceived privacy risk regarding wearable health technology was measured using 3 items [65], each rated on a 7-point Likert scale (1=strongly disagree to 7=strongly agree; eg, “It would be risky to disclose my personal health information to wearable device vendors”).

#### Health Consciousness

Health consciousness was measured using a 3-item scale, with participants indicating their agreement with each statement on a 7-point Likert scale (1=strongly disagree to 7=strongly agree; eg, “I take responsibility for the state of my health”) [66].

#### Self-Determination Factors

Adapted from Ryan et al [67], 3 factors of self-determination—competence, autonomy, and relatedness—were measured using a 7-point Likert scale (1=strongly disagree to 7=strongly agree). Competence in using wearable health devices was measured using a 3-item scale (eg, “I would feel competent in using a smart wearable health care device”). Autonomy was assessed with a 4-item scale (eg, “I would feel free to decide for myself how to do things on a smart wearable health care device”). Relatedness to other users of wearable health care devices was measured using 3 items (eg, “I would find the relationships with other users of smart wearable health care devices fulfilling”).

#### Future Use Intention

Intention to adopt smart wearable health care technology was measured using a 3-item semantic differential scale. Participants rated their likelihood, probability, and willingness to use smart wearable health care technology in the next 3 months, with responses ranging from 1 to 7 (eg, likelihood: 1=unlikely to 7=likely).

Refer to Table 1 for the measurement items, internal reliability (Cronbach α), and basic statistics for each factor’s composite score.

**Table 1.** Measurement items, internal reliability, and descriptive statistics.

Measures and items			Cronbach α	Mean (SD)
AI^a^ anxiety			0.78	3.95 (1.28)
	I feel apprehensive about using AI technology.			
	I hesitate to use AI technology for fear of making mistakes I cannot correct.			
	I have difficulty understanding AI-related technological matters.			
Perceived privacy risk			0.92	4.40 (1.44)
	It would be risky to disclose my personal health information to wearable device vendors.			
	There would be a high potential for loss associated with disclosing my personal health information to vendors providing wearable devices.			
	There would be too much uncertainty associated with giving my personal health information to vendors providing wearable devices.			
Health consciousness			0.63	5.37 (1.06)
	I am concerned about my health all the time.			
	I notice how I feel physically as I go through the day.			
	I take responsibility for the state of my health.			
Competence			0.95	4.83 (1.31)
	I would feel competent in using a smart wearable health care device.			
	I would feel capable when using a smart wearable health care device.			
	I would feel like I am effective when using a smart wearable health care device.			
Autonomy			0.91	4.70 (1.17)
	I would feel free to decide for myself how to do things on a smart wearable health care device.			
	I would generally feel free to express my ideas and opinions on a smart wearable health care device.			
	I would feel like I can pretty much be myself when I use a smart wearable health care device.			
	I would experience a lot of freedom when I use a smart wearable health care device.			
Relatedness			0.70	4.03 (.97)
	I would find the relationships with other users of smart wearable health care devices fulfilling.			
	I would find the relationships with other users of smart wearable health care devices important.			
	I would not feel close to other smart wearable health care device users (reverse-coded).			
Intention			0.94	4.78 (1.93)
	How likely are you to use smart wearable health care devices in the next 3 months?			
		Unlikely or likely (1 to 7 scale)		
		Not probable or probable (1 to 7 scale)		
		Unwilling or willing (1 to 7 scale)		

a AI: artificial intelligence.

### Measurement Model

The measurement model was tested using comparative fit index (CFI) analysis. Covariances for the first 3 autonomy items and the first 2 competence items were allowed to improve model fit. After this modification, the model showed good fit: *χ*²_185_=365.9, *P*<.001, root mean square error of approximation (RMSEA)=0.057 (90% CI 0.048-0.065), CFI=0.966, Tucker-Lewis index (TLI)=0.957, and standardized root mean square residual (SRMR)=0.048, all within acceptable ranges.

## Results

The structural equation model (SEM) results indicate significant associations among AI anxiety, perceived privacy risks, and health consciousness, and the mediating factors of competence, autonomy, and relatedness, which are related to smart health wearable use intention. Model fit indices suggested a good fit: *χ*²_226_=541, *P*<.001; RMSEA=0.067 (90% CI 0.06-0.075); CFI=0.94; TLI=0.93; SRMR=0.06.

For RQ1, AI anxiety was negatively associated only with competence (β=−.25, *P*=.039); perceived privacy risks showed a significant negative association with competence (β=−.33, *P*=.002), autonomy (β=−.3, *P*=.012), and relatedness (β=−.36, *P*=.001). Health consciousness was positively associated with competence (β=.78, *P*<.001), autonomy (β=.8, *P*<.001), and relatedness (β=.61, *P*<.001).

**Figure 2.**
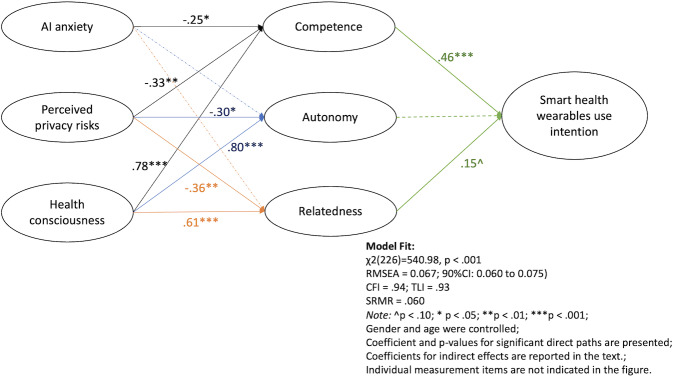
Structural equation model results for RQ1 and H1. AI: artificial intelligence; CFI: comparative fit index; H: hypothesis; RMSEA: root mean square error of approximation; RQ: research question; SRMR: standardized root mean square residual.

H1 examined the associations between the 3 self-determination model dimensions and the intention to use smart health wearables. Competence was significantly associated with use intention (β=.46, *P*<.001), and relatedness also showed a marginally significant association (β=.15, *P*=.05). Figure 2 summarizes the SEM results.

To answer RQ2, we examined the indirect effects of AI anxiety, perceived privacy risks, and health consciousness on use intention through competence, autonomy, and relatedness as mediators, using 5000 bootstrapped samples. The total indirect effect of AI anxiety on use intention was not statistically significant (*B*=−0.31, 95% CI −0.78 to 0.05) although the indirect pathway through competence was significant in a negative direction (*B*=−0.27, 95% CI −0.71 to −0.05). For perceived privacy risks, the total indirect effect was significant in a negative direction (*B*=−0.36, 95% CI −0.59 to −0.14), via competence (*B*=−0.24, 95% CI −0.52 to −0.08) and relatedness (*B*=−0.09, 95% CI −0.23 to −0.004). Lastly, health consciousness showed a significant total indirect effect on use intention in a positive direction (*B*=1.37, 95% CI 1.01-2.08), with a strong positive mediation through competence (*B*=0.97, 95% CI 0.44-1.78) and relatedness (*B*=0.25, 95% CI 0.02-0.58). We found no significant indirect pathways through autonomy for any predictors.

Finally, RQ3 asked if experienced versus not experienced older adult users would have different motivation structures shaping the intention to use smart health wearables. First, measurement invariance between experienced versus nonexperienced users for the 3 self-determination factors and use intention were tested. The models allowing for free estimation of all parameters across groups showed a good fit to the data: *χ*^2^_110_=183.8, *P*<.001; RMSEA=0.066, 90% CI 0.049-0.083; CFI=0.979; TLI=0.971; and SRMR=0.045. Additionally, the chi-square difference test comparing the model constraining the factor loadings to be equal across groups, against the unconstrained model, was not significant (Δ*χ*^2^_9_=6.8, *P*=.66), indicating that the factor loadings are invariant across groups. To assess the relationship between the 3 motivation factors and behavioral intention across experienced and nonexperienced user groups, 2 SEMs were estimated: an unconstrained path coefficient model (*χ*^2^_18_=38.5, *P*=.003, RMSEA=0.086, 90% CI 0.048-0.124, CFI=0.968) and a constrained model (*χ*^2^_30_=50.5, *P*=.011, RMSEA=0.067, 90% CI 0.032-0.098, CFI=0.968), both showing acceptable model fits. However, the chi-square difference test indicated that the relationships between competence, autonomy, and relatedness, and behavioral intention between the 2 groups do not statistically differ (Δ*χ*^2^_12_=12, *P*>.05).

## Discussion

### Theoretical Implications

Drawing on SDT, this study sheds light on the motivational factors influencing older adults’ intention to use smart wearables for health care. By examining external contextual factors that may shape older adults’ autonomous use of smart wearables for health management, we provide more nuanced insights into the self-determination model, enhancing the understanding of how to promote self-determined, proactive health management through technology. Moreover, this age-specific insight contributes to a deeper understanding of how SDT can be adapted to different user populations in the smart health wearables context.

Our study examined how external contextual factors influence intrinsic motivation, particularly in the adoption of smart health wearables among older adults (RQ1). The results reveal that perceived privacy risks erode all motivation factors: competence, autonomy, and relatedness. The fear of data breaches or the misuse of personal health information can make older adults feel that they lack control over their personal data, directly undermining their sense of autonomy. Perceived privacy risks also affect competence, as older adults may feel uncertain about their ability to navigate complex privacy settings or how their data are collected and used. This uncertainty creates a barrier to engaging with smart health wearables, as users who feel less competent in managing their personal information may be less likely to adopt such technologies. The negative association between privacy risk perception and relatedness may imply that fears of data misuse or unauthorized access can undermine users’ willingness to engage in social features of smart health wearables, such as sharing health information with peers or participating in health communities. We also found a significant association between AI anxiety and competence, among 3 intrinsic motivation factors. AI anxiety is characterized by fear and apprehension about AI’s decision-making processes [8]. Older adults, who may already feel less confident in their technological abilities, may find AI’s opaque algorithms intimidating, leading them to doubt their capacity to effectively use smart health wearables, consequently reducing their intrinsic motivation to use them.

However, health consciousness strongly supports the fulfillment of all 3 psychological needs among older adult users; health-conscious older adults are more likely to feel competent, autonomous, and socially connected through the use of smart health wearables. This result indicates that the tendency of being proactive in self-health management among health-conscious individuals [47,50] can be largely translated to self-determined smart health wearables adoption. Especially, the significant association between health consciousness and relatedness suggests that older adults who care about their health are more likely to engage with smart health wearables and connect with others through health communities and shared goals, not just for self-health management. These findings suggest that while SDT offers a strong framework for understanding motivation across various domains, it may require adjustments for technology adoption among older adults.

We then examined the associations between the 3 psychological needs that foster intrinsic motivation and the intention to use smart health wearables (H1). The results indicate that, for older adults, feeling capable of using smart health wearables (competence) is a significant driver of wearable use, and experiencing social connection (relatedness) showed a marginally significant association. However, independent decision-making (autonomy) was not a significant factor. This result might be because older adults recognize the benefits of delegating monitoring and decision-making for health management to technology, which provides personalized recommendations for users. This result suggests that older adults may prioritize supported autonomy in using smart health wearables. Studies [29,68] have suggested that users seek to benefit from the autonomous capacity of AI-based technology while maintaining control over it.

It is also worth noting that our result contradicts Jung and Kang [52], who found that only autonomy, among the 3 intrinsic motivation factors, significantly predicted enjoyment in using smart fitness wearables, based on a survey conducted with a general population in the United States. This also indicates a possibility that autonomy might be experienced or expected differently among the older population in Singapore. Future research should explore how older adults balance autonomy and supported autonomy through health technologies and how smart health wearables can foster this balance without diminishing their sense of agency, especially through comparison with young users.

The mediating roles of the 3 psychological needs between contextual factors and the intention to use smart health wearables (RQ2) offer a deeper understanding of the factors influencing self-determination in wearable health technology use. The findings underscore the importance of fostering feelings of competence to encourage smart wearable adoption among older adults. Competence, as a key driver of intrinsic motivation, is particularly crucial for this demographic, who may feel intimidated or uncertain about using such technology [54]. The strong indirect associations between privacy risk perceptions, health consciousness, and usage intentions through competence suggest that merely addressing privacy concerns and promoting health awareness may be insufficient. Instead, efforts should also focus on clearly communicating privacy safeguards and emphasizing the value of self-directed health management in a way that reinforces older adults’ confidence and competence, ultimately helping them integrate wearables into their health routines successfully.

While relatedness played a secondary role, it nonetheless contributes to older adults’ intrinsic motivation, aligning with previous research identifying relatedness as an important intrinsic motivator in social media use [69]. Our study indicates that the feelings of being connected to peer users, family, and caregivers remain a significant need for older adult users even when engaging with technology primarily intended for purposes other than social interaction or relationship building.

Finally, the lack of significant differences between experienced and nonexperienced users regarding the associations between motivational factors and use intention (RQ3) suggests that the need for competence and relatedness is important in shaping the intention to adopt smart health wearables, regardless of prior experience with such devices. Previous research found that individuals with prior experience using smart devices (eg, smartphones and smart televisions) perceived them as less complex and were more willing to try new technologies, as compared to those without experience [19]. However, the absence of differences between the 2 groups in our study could be interpreted in 2 ways. First, advancements in user-centered design and AI may have made smart health wearables more intuitive and less cognitively demanding [18], reducing the potential barriers or concerns around competence in using these devices. Alternatively, it is possible that smart health wearables remain complex and intimidating, even for older adults who have had prior experience with similar technology. Future research could use a qualitative approach, such as in-depth interviews with older adult users, to explore the underlying reasons behind this nonsignificant result. In addition, for older adult users, connecting with family and friends was identified as a major gratification in using new media technology [69-71]. Our study demonstrates that, for both experienced and nonexperienced groups, fostering relatedness with others remains an important reason for adopting health wearables among older adults, which often did not hold truth in other populations [24,52].

### Practical Implications

Our findings indicate that perceived privacy risks can negatively impact older adults’ sense of competence and autonomy when adopting smart health wearables. To address perceived privacy risks, smart health wearables should offer easy-to-use privacy settings that give older adults control over how their data are shared. Transparent communication about data security can enhance users’ sense of control and trust, encouraging wider adoption and sustained use.

Competence emerged as the strongest predictor of smart health wearables adoption. Therefore, developers should focus on creating intuitive, easy-to-use devices tailored to older adults, with guides, tutorials, and support resources that enhance users’ confidence and ability. Features such as personalized feedback, goal-setting, and progress tracking can further reinforce older adults’ sense of accomplishment in managing their health, fostering continued engagement. By helping older adults feel capable and effective, developers can promote long-term use of wearables.

The feeling of social connectedness (relatedness) also plays a significant role in driving smart health wearables adoption. Developers should incorporate social features that allow older adults to connect with family, friends, or health communities—such as shared health data, group challenges, and support groups. These features foster a sense of social support, enhancing motivation. Health care providers and caregivers can encourage older adults to use smart health wearables in social settings, which can strengthen their motivation for ongoing use.

The nonsignificant difference between experienced and nonexperienced users in terms of the motivational structure for smart health wearables use suggests that both groups rely on feeling competent and socially connected when deciding to use smart health wearables. From a practical standpoint, this indicates that interventions aimed at enhancing these motivational factors—such as simplifying the technology to improve competence and fostering social connections—are equally important for both groups. For example, social workers and government organizations can provide training courses for older adults focusing on these aspects. A recent study showed that even short training courses (eg, teaching older adults how to use smartphones for social interaction and medical use) increased the positive effect of older adult users’ effort expectancy (how understandable and easy to learn to use the technology) on behavior intention [72]. Therefore, strategies to increase smart health wearables adoption should focus on addressing these core needs universally, rather than differentiating between experienced users and nonexperienced users.

### Limitations and Future Research

We acknowledge several limitations in this study. First, our cross-sectional design limits the ability to establish causal relationships between autonomy-related factors and wearables adoption. As a result, the associations observed in this study are correlational. Longitudinal research would be valuable in examining how variables such as AI anxiety, privacy concerns, and health consciousness impact both short- and long-term use of wearables over time, providing a clearer understanding of causality.

Second, this study was conducted via a web-based survey, which inherently requires participants to have a certain level of digital literacy and internet access. Consequently, our sample may not fully represent older adults with limited internet access or low digital literacy. To capture a more inclusive profile of older adult participants, future studies should consider using offline methods, such as paper-based surveys, to ensure a broader representation of older adults with varying digital skills.

Third, while our research primarily focuses on intrinsic motivation among older adult users for adopting smart health wearables, actual behavioral intention (ie, intention to use) is also influenced by external factors, such as the usability designs of the devices. Studies indicate that older adults have specific performance expectations regarding wearable functionality and ease of use [73]. Therefore, future studies should consider both intrinsic and external factors when exploring predictors of older adults’ adoption and sustained use of smart health wearables.

Fourth, the high ownership rate of fitness trackers among our participants (135/306, 44.1%) can be attributed to Singapore’s National Steps Challenge, initiated in 2015, which provided free fitness trackers to encourage physical activity. This unique context may have influenced our results, highlighting the need for further cross-cultural studies in other countries to gain complementary insights and verify the generalizability of these findings across different cultural and health care contexts.

Finally, according to the National Population and Talent Division, 81.8% of those over 60 years old are ethnic Chinese, 11% are Malay, 8% are Indian, and 1% belong to other ethnicities as of 2024 [74]. While our sample largely reflects the overall ethnic group composition, Chinese (259/306, 84.6%) and other ethnicities (17/306, 5.6%) are slightly overrepresented, and Malay (10/306, 3.3%) and Indian participants (20/306, 6.5%) are slightly underrepresented. Given that cultural and ethnic differences could significantly influence health-related [75] as well as technology and privacy-related factors [76,77], future research should aim for a more representative sample to capture the diverse experiences and preferences of all ethnic groups, ensuring broader applicability of the findings.

### Conclusions

Through the lens of SDT, this study advances our understanding of older adults’ adoption of smart health wearables. The results highlighted the critical roles of competence, relatedness, and autonomy-supporting contexts in shaping their intrinsic motivation. While perceived privacy risks and AI anxiety negatively impact motivation, health consciousness emerges as a strong enabler of self-determined adoption of health wearables. Our findings emphasize the need for user-friendly designs, robust privacy safeguards, and social features that foster connection and confidence among older adults. By addressing these factors, developers and stakeholders can better support older adults in integrating smart wearables into their proactive health management routines.

## Abbreviations

AI: artificial intelligence
CFI: comparative fit index
H: hypothesis
RFID: radio frequency identification
RMSEA: root mean square error of approximation
RQ: research question
SDT: self-determination theory
SEM: structural equation model
SRMR: standardized root mean square residual
TAM: technology acceptance model
TLI: Tucker-Lewis index
UTAUT: Unified Theory of Acceptance and Use of Technology
